# Ectodermal Dysplasia Presenting as Hypodontia in a Nine-Year-Old Female

**DOI:** 10.7759/cureus.26806

**Published:** 2022-07-13

**Authors:** Brenda Abreu Molnar, Alejandro Semidey, Suzanne Minor, Katherine Semidey

**Affiliations:** 1 Medicine, Florida International University, Herbert Wertheim College of Medicine, Miami, USA; 2 Dentistry, University of Florida, Miami, USA; 3 Family Medicine, Florida International University, Herbert Wertheim College of Medicine, Miami, USA; 4 Pediatrics, Florida International University, Herbert Wertheim College of Medicine, Miami, USA

**Keywords:** pediatric case report, social determinants of health (sdoh), abnormal teeth, hypodontia, ectodermal dysplasia

## Abstract

Ectodermal dysplasia describes a group of disorders that involve abnormal development of ectodermal tissue, including hair, teeth, and sweat glands. This report presents a case of a child affected by ectodermal dysplasia presenting as abnormal teeth development. Social determinants of health, including recent immigration from an underserved area and lack of funds, have limited this child from an earlier diagnosis and have formed barriers to access proper oral rehabilitation.

## Introduction

A 9-year-old female presented for a well-child visit. Physical examination revealed abnormal dentition that suggested ectodermal dysplasia. The objective of this case report is to highlight the presentation of this rare condition in the setting of barriers to care. Current literature contributes to the evolving classification of ectodermal dysplasia, discusses the variety in presentation, and explores the importance of dental management in improving quality of life.

Ectodermal dysplasia describes a group of over 100 genetic disorders that heterogeneously affect the normal development of ectodermal tissue. Patients present with abnormalities in the integumentary and nervous systems, contributing to the diversity of disease presentation [1]. Hypodontia, or congenitally missing teeth, is a common presentation that sparked our initial inquiry with our patient.

The Freire-Maia and Pinheiro classification system, developed in 1982, initially categorized these diseases clinically by their involvement of the teeth, hair, nails, or sweat glands [2]. They were subcategorized as “Group A” and involved at least two of the aforementioned tissues, or “Group B” and affected only one of these tissues with at least one other tissue of ectodermal origin [1]. The most recent system developed in 2019 at the National Institutes of Health, integrates clinical and molecular information in classifying ectodermal dysplasias [2]. Various genes have been discovered to play a role in the pathogenesis of alterations in ectodermal tissue, including EDA and WNT10A. In particular, WNT10 variants result in missing teeth, but with no other phenotypic features of ectodermal dysplasia [2]. The most common inherited form of the disease is X-linked hypohidrotic ectodermal dysplasia, which classically presents with hypodontia, reduced ability to sweat (hypohidrosis), and sparse hair (hypotrichosis) [3]. Autosomal dominant and autosomal recessive inheritance patterns have also been identified for other forms of the condition [4]. Despite over 170 forms of ectodermal dysplasia being identified, it is an uncommon disease, with an incidence of 1 in 100,000 births [4].

Management of ectodermal dysplasia is normally targeted at treating the symptoms of each unique presentation. When treating abnormal dentition, providers must consider masticatory dysfunction and commonly associated lower salivary flow rate and decreased buffering capacity, meaning patients are at a far greater risk of decay. Therefore, early preventive treatment, such as meticulous oral hygiene instruction, regular fluoride use, and xylitol is very helpful in decreasing the risk for decay [5]. However, the issue of aesthetics must be seriously addressed due to the high potential for psychosocial difficulties, low self-esteem, and social isolation during school years, adolescence, and beyond. In the case of hypodontia or anodontia, the most common management plan involves a removable prosthesis, which improves mastication, articulation, and self-esteem [4]. Although implants are a restorative option, they offer a decreased success rate of implant survival compared to nonaffected patients. This appears to be associated with the available maxillary and mandibular bone volume in ectodermal dysplasia patients due to a lack of development of permanent dentition. This does not preclude patients from receiving dental implant treatment, but it does compromise the ideal positioning of the implants and subsequent long-term success [6].

## Case presentation

A healthy 9-year-old female who recently immigrated from Venezuela by way of Colombia presented to a free healthcare clinic for a well-child visit. The mother had concerns about her daughter’s chronic cough (which she attributed to environmental allergies), abnormal teeth development, and family history of thyroid diseases (for which she desired thyroid function blood testing).

According to the mother, the patient’s primary central incisors shed at 6 to 7 years of age. Although her primary teeth were normally shaped, her permanent dentition erupted with areas of missing teeth, as well as small and abnormally shaped anterior maxillary and mandibular teeth. Her mother noted that other children called her daughter’s teeth “shark teeth,” causing emotional distress for her in school. Her mother wondered if the teeth erupted abnormally secondary to improper supplementation or nutrition during her childhood. She was seen by a dentist in Colombia where a panoramic radiograph was taken, showing anterior unerupted teeth, however, she never received a diagnosis. There was no history of complications for the child at birth, although her mother did have preeclampsia requiring a C-section. The patient has no other associated deformities or abnormalities, and no other family members were identified as having similar teeth findings. However, the mother noted that the patient’s paternal aunt was born with contractures or abnormal positioning of the fingers.

**Figure 1:**
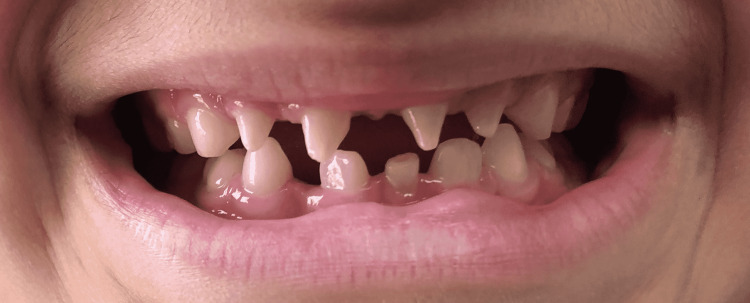
Intraoral profile view of the 9-year-old female showing tapered maxillary central and lateral incisors with open spaces between the teeth.

On physical exam, the patient demonstrated no signs of hypohydrosis or hypotrichosis. Intraoral examination revealed four tapered maxillary central and lateral incisors with open spaces between teeth (Figure 1). The mandibular central incisors were also spaced out, with peg-shaped and flattened edges. Overall, the incisors were smaller than the pre-molars and molars.

## Discussion

This patient’s case clearly demonstrates a child with hypodontia requiring diagnosis and management. The differential diagnosis for hypodontia includes ectodermal dysplasia, facial trauma, teratogenic drug use during pregnancy, or congenital rubella [7]. This patient’s presentation of hypodontia along with microdontia and tapering teeth, in the absence of known trauma or infections and teratogen use during pregnancy, make a form of ectodermal dysplasia the most likely diagnosis. Although testing is available for gene analysis or by biopsy and histology, a clinical diagnosis is also considered appropriate, particularly in cases such as this, where barriers to care are present.

This case is similar to physical exam findings in various case reports of ectodermal dysplasia, in which patients had conical, peg-shaped teeth with areas of missing dentition [4]. Based on prior case reports, positive outcomes of improved self-confidence and masticatory function, it would be recommended for this patient to seek assistance from a multidisciplinary team of dentists, geneticists, and a social worker to help navigate socioeconomic barriers.

The patient has overcome various social adversities, including emigrating from Venezuela due to political turmoil, seeking refuge in Colombia, and finally arriving in the United States to stay with a family member while applying for political asylum. Without health insurance, she has also had limited access to healthcare facilities, seeking care in free clinics. Despite having a panoramic radiograph completed in Colombia, she was never given an official diagnosis and was unable to treat her teeth deformities due to high out-of-pocket costs.

Access to a multidisciplinary team may be achieved by enrolling this family in local non-profit organizations, such as the Green Family Foundation Neighborhood Health Education Learning Program at the Florida International University Herbert Wertheim College of Medicine. This program consists of interprofessional teams of students and professionals in various disciplines such as medicine, nursing, social work, education, and law, who address needs identified by enrolled households [8]. National organizations, such as the National Foundation for Ectodermal Dysplasia, which offers treatment assistance programs for low-income patients, may also be an option for this case [9].

## Conclusions

Ectodermal dysplasia describes a group of genetic disorders presenting with abnormal development in ectodermal tissue, including teeth, hair, and sweat glands. This was a case of a 9-year-old female who presented for a well-child visit and was discovered to have possible ectodermal dysplasia presenting as hypodontia with abnormally shaped and tapering teeth. This paper adds to the scientific literature on an uncommon condition and can give providers insight into the variability of disease presentation. Further evaluation for this patient will be required to determine orthodontic solutions for her teeth abnormalities. Coupled with financial and immigration barriers, this patient will require support from the community and national programs to provide options to address this rare condition and improve her quality of life.
